# pHluorin-BACE1-mCherry Acts as a Reporter for the Intracellular Distribution of Active BACE1 In Vitro and In Vivo

**DOI:** 10.3390/cells8050474

**Published:** 2019-05-17

**Authors:** Lu Zhao, Yang Zhao, Fu-Lei Tang, Lei Xiong, Ce Su, Lin Mei, Xiao-Juan Zhu, Wen-Cheng Xiong

**Affiliations:** 1 Key Laboratory of Molecular Epigenetics of Ministry of Education, Institute of Cytology and Genetics, Northeast Normal University, Changchun 130024, China; zhaol014@nenu.edu.cn (L.Z.); yxz1956@case.edu (Y.Z.); suc7441@126.com (C.S.); 2 Department of Neurosciences, School of Medicine, Case Western Reserve University, Cleveland, OH 44106, USA; lxx156@case.edu (L.X.); lxm387@case.edu (L.M.); 3 Department of Neuroscience and Regenerative Medicine, Medical College of Georgia, Augusta University, Augusta, GA 30912, USA; ftang@augusta.edu

**Keywords:** pHluorin-BACE1-mCherry, BACE1, intracellular trafficking, pH regulation, PRR, APP, Alzheimer disease

## Abstract

β-site APP-cleaving enzyme 1 (BACE1) initiates amyloid precursor protein (APP) cleavage and β-amyloid (Aβ) production, a critical step in the pathogenesis of Alzheimer’s disease (AD). It is thus of considerable interest to investigate how BACE1 activity is regulated. BACE1 has its maximal activity at acidic pH and GFP variant—pHluorin—displays pH dependence. In light of these observations, we generated three tandem fluorescence-tagged BACE1 fusion proteins, named pHluorin-BACE1-mCherry, BACE1-mCherry-pHluorin and BACE1-mCherry-EGFP. Comparing the fluorescence characteristics of these proteins in response to intracellular pH changes induced by chloroquine or bafilomycin A1, we found that pHluorin-BACE1-mCherry is a better pH sensor for BACE1 because its fluorescence intensity responds to pH changes more dramatically and more quickly. Additionally, we found that (pro)renin receptor (PRR), a subunit of the v-ATPase complex, which is critical for maintaining vesicular pH, regulates pHluorin’s fluorescence and BACE1 activity in pHluorin-BACE1-mCherry expressing cells. Finally, we found that the expression of Swedish mutant APP (APPswe) suppresses pHluorin fluorescence in pHluorin-BACE1-mCherry expressing cells in culture and in vivo, implicating APPswe not only as a substrate but also as an activator of BACE1. Taken together, these results suggest that the pHluorin-BACE1-mCherry fusion protein may serve as a useful tool for visualizing active/inactive BACE1 in culture and in vivo.

## 1. Introduction

BACE1 (β-site APP-cleaving enzyme 1), also called β-secretase, initiates amyloid precursor protein (APP) cleavage, a critical step for β-amyloid (Aβ) production [1,2,3]. Aβ is believed to be a critical detrimental factor for the pathogenesis of Alzheimer’s disease (AD), the most common form of dementia affecting 10% of all people over 65 years of age [4,5,6]. *App* is a Mendelian gene for early-onset AD. App mutations (e.g., Swedish mutations) identified in the early onset AD patients promote the generation of Aβ by favoring proteolytic processing of APP by β-secretase [7,8,9]. Overexpression of BACE1 increases β-secretase cleavage of APP and Aβ generation and BACE1 knock-out prevents Aβ production [10,11,12]. Thus, significant efforts have been made to understand how BACE1 activity is regulated.

BACE1, a member of the peptidase A1 family of aspartic proteases, contains an N-terminal signal peptide (SP) (residues 1–21), a pro-peptide (Pro) domain (residues 22–45), a catalytic domain (residues 46–454), a transmembrane domain (residues 455–478) and a C-terminal tail (residues 479–501). The signal peptide and Pro domain are removed posttranslationally, resulting in the mature BACE1 enzyme beginning at residue Glu46 [13]. BACE1 has two aspartic protease active site motifs, DTGS (Asp-Thr-Gly-Ser)(residues 93–96) and DSTG (Asp-Ser-Thr-Gly)(residues 289–292) and mutation of either aspartic acid renders the enzyme inactive [7,13]. In addition, BACE1′s single transmembrane domain is near its C terminus, which can be palmitoylated [14,15,16].

BACE1 is believed to cleave APP primarily in early or late endosomes because BACE1′s protease activity is optimal in the acidic environment of endosomal compartments [17,18,19,20,21]. The Aβresulting from β- and γ-secretase cleavage can then be released into the extracellular space, likely by exosomes [22,23,24]. Therefore, investigating how BACE1 trafficking is regulated has a significant impact on our understanding of BACE1 activation/inactivation and Aβ production. BACE1 trafficking occurs along the constitutive secretory pathway to the cell surface. BACE1 is initially synthesized in the endoplasmic reticulum (ER) as an immature precursor protein (proBACE1) [25,26,27,28]. Short-lived proBACE1 undergoes rapid maturation in the trans-Golgi network (TGN), where the propeptide is removed by proteolytic cleavage using furin or furin-like convertases [25,26,29], and complex carbohydrates are added. The mature form of BACE1 traffics from the TGN to the plasma membrane, where a small proportion can undergo ectodomain shedding, which is suppressed by palmitoylation [14]. The majority of BACE1 at the plasma membrane undergoes internalization into endosomes, where the acidic environment provides the optimal conditions for the proteolysis of APP [25,28,30,31]. Endosomal BACE1 can be recycled back to the cell surface [28,32,33], transit to lysosomes for degradation [34] and return to the TGN through retrograde transport [32,35,36,37].

To investigate BACE1 trafficking and activation between intracellular vesicles, fluorescence imaging of live cells is the most practical approach because it offers adequate spatiotemporal resolution under physiological conditions. We generated a dual-fluorescence-based BACE1 reporter, in which BACE1 is fused with the pH-sensitive green fluorescent protein (GFP) variant pHluorin (as a reporter for inactive BACE1) and the pH-stable red fluorescence protein mCherry (as a marker for BACE1 distribution and expression). It is our hope that this pHluorin-BACE1-mCherry fusion protein can be a useful tool to visualize active/inactive BACE1 trafficking in cultured cells and in vivo.

## 2. Materials and Methods

### 2.1. Animals

Mice were housed in a room with a 12 h light/dark cycle with water and a rodent chow diet. Females of the indicated mouse strains were bred overnight with males. The noon after breeding when a vaginal plug was found was considered embryonic day 0.5 (E0.5) and the day of birth was considered postnatal day 0 (P0). Experiments were replicated at a minimum of three times with mice derived from independent litters.

The floxed (pro)renin receptor (PRR) mice (PRR^f/f^) were kindly provided by Dr. Katrina J. Binger (Experimental and Clinical Research Center, Berlin, Germany) and described previously [38]. The LSL-APPswe mice were also described previously [39]. In this mouse line, APPswe protein expression is under the control of the cytomegalovirus (CMV) promoter but its protein expression is blocked by a loxP-stop-loxP and requires Cre mediated recombination.

All animal experiments were approved by the Institutional Animal Care and Use Committee of Case Western Reserve University, USA, according to the National Institutes of Health (NIH) guidelines.

### 2.2. Antibodies

Primary antibodies used in this project and their final concentrations were as follows: anti-GFP (Life technology, Carlsbad, CA, USA, 1:1000), anti-RFP (Rockland, Limerick, PA, USA, 1:1000), anti-β-actin (Sigma-Aldrich, St. Louis, MO, USA, 1:5000), anti-GM130 (BD, Franklin Lakes, NJ, USA, 1:500), anti-EEA1 (BD, Franklin Lakes, NJ, USA, 1:500), anti-Rab 7 (Santa Cruz, Santa Cruz, CA, USA, 1:500), anti-LAMP1 (DSHB, Iowa, IA, USA, 1:500), anti-PRR/ATP6AP2 (Sigma-Aldrich, St. Louis, MO, USA, 1:500), anti-APP (Cell Signaling Technology, Danvers, MA, USA, 1:500), anti-sAPPβ (a gift from Tae-Wan Kim, 1:500). All the corresponding conjugated secondary antibody (1:1000) were purchased from Invitrogen (Waltham, MA, USA). Nuclei were stained with 4′,6-diamidino-2-phenylindole (DAPI) (1:1000, Roche, Basel, Switzerland).

### 2.3. Expression Plasmids

pHluorin was inserted into the end of the BACE1-mCherry pro-peptide by ligation independent cloning (LIC) with Exonuclease III to generate the pHluorin-BACE1-mCherry plasmid. Then, the ORF with pHluorin-BACE1-mCherry was inserted into a mammalian expression vector under the control of the CAG promoter. To generate the BACE1-mCherry-pHluorin plasmid, we first inserted BACE1-mCherry from BACE1-mCherry-IRES-EGFP into a mammalian expression vector under the control of the CAG promoter using AscI and ClaI sites. Next, we amplified pHluorin from pCMV-lyso-pHoenix (Addgene, Catalog #70112) by PCR. Finally, pHluorin was inserted C-terminal of the mCherry using NotI and XhoI sites. The BACE1-mCherry-EGFP plasmid was generated by amplifying BACE1-mCherry from BACE1-mCherry-IRES-EGFP by polymerase chain reaction (PCR) and subcloning it into the pEGFP-N1 (Clontech Laboratories, Catalog #6085-1) mammalian expression plasmid using SacI and BamHI sites.

The CAG-BFP plasmid is described previously [40]. The V5-PRR plasmid was purchased from DNASU (ATP6AP2 in pLX304, HsCD00446844) and the CMV promoter was replaced with the CAG promoter. The pCAG-cre and pCAX-Flag-APP plasmids were purchased from Addgene. The authenticity of all constructs was verified by DNA sequencing.

### 2.4. Western Blot Analysis

HEK293 cells transfected with pHluorin-BACE1-mCherry, BACE1-mCherry-pHluorin or BACE1-mCherry-EGFP were lysed in lysis buffer (50 mM Tris-HCl (pH ~7.4), 150 mM NaCl, 1% NP-40, 0.5% Triton X-100, 1 mM phenylmethylsulfonyl fluoride (PMSF), 1 mM EDTA, 5 mM sodium fluoride and 2 mM sodium orthovanadate) containing protease inhibitor cocktail (Roche). Samples were incubated with lysis buffer for 20 min on ice, the cell suspension was transferred to a microcentrifuge tube, oscillated every 5 min and then centrifuged at 12,000× *g* for 15 min. The supernatant was measured by a BCA (Thermo Fisher, Waltham, MA, USA) assay. A total of 10–20 µg protein was loaded into 10% SDS-PAGE gels to separate proteins. The proteins in the gels were transferred onto a nitrocellulose membrane (Bio-Rad, Hercules, CA, USA). After electrotransfer, the nitrocellulose membranes were blocked in 5% bovine serum albumin (BSA) for 1 h at room temperature. Antigen-specific primary antibodies were diluted to the proper concentrations and incubated overnight at 4 °C. The membranes were washed 3 times and incubated with a secondary antibody (1:5000, Thermo Fisher) for 1 h at room temperature. We used a chemiluminescent horseradish peroxidase (HRP) antibody detection kit (HyGLO^TM^ Quick Spray) for visualization of the signal.

### 2.5. Cell Culture and Transient Transfection

NLT (a GnRH neuroblastoma cell line) or MC3T3-E1 cells were maintained in Dulbecco’s modified Eagle’s medium (DMEM) supplemented with 10% fetal calf serum (FBS) and 100 units/mL penicillin G and streptomycin (Gibco, Waltham, MA, USA). Primary dissociated cortical neuronal cultures were prepared from E18.5 mouse embryos as previously described [41,42]. Briefly, dissociated cortical neurons were plated on 35 mm poly-d-lysine-coated glass-bottom Microwell dishes (MatTek) at a density of 50,000 cells/cm^2^ and were maintained in Neurobasal medium (Invitrogen) containing B-27 plus (Gibco) and 2 mM GlutaMAX (Gibco).

For transfection, NLT cells were plated at a density of 10,000 cells/cm^2^ in a 35 mm glass-bottom Microwell dishes and allowed to grow for 12 h before transfection using polyethylenimine (PEI). Forty-eight hours after transfection, the cells were subjected to live cell imaging analysis. Neurons were transfected with various constructs at DIV4 using the calcium phosphate method, followed by live cell imaging analysis at DIV6 and immunostaining analysis at DIV7, as described previously [41,43].

The PRR-KD cell line was obtained as described previously [44]. In brief, MC3T3-E1 cells were infected with shRNA-PRR lentiviral particles in medium containing 2 µg/mL polybrene. After 24 h, the culture medium was removed and replaced with 10% FBS DMEM (without polybrene). Stable clones expressing shRNA-PRR were selected with 5 µg/mL puromycin dihydrochloride after 5–6 days.

### 2.6. BACE1 Activity Measurement

BACE1 activity was measured using its detection kit (CS0010; Sigma-Aldrich) according to the manufacturer’s instructions. In brief, HEK293 cells transfected with indicated plasmids were lysed in lysis buffer (50 mM Tris, pH 7.5, 150 mM NaCl, 5 mM EDTA, 1% Triton X-100, 0.1% sodium deoxycholate) without protease inhibitors and centrifuged at 12,000 rcf for 15 min. The supernatant was measured by a BCA (Thermo Fisher) assay to determine the protein concentration. A total of 140 µg protein was loaded per well in a white 96-well plate with 50 µM BACE1 substrates. Immediately after the addition of BACE1 substrates, samples were placed in a fluorescent plate reader (SynergyHTX; Biotek, Winooski, VT, USA) and maintained at 37 °C. Fluorescence was measured every 5 min for 180 min with excitation at 320 nm and emission at 405 nm. The percentage of BACE1 substrate cleavage was calculated based on a calibration curve with standard samples.

### 2.7. ELISA Analysis of Aβ (1–40)

Aβ (1–40) levels were measured using a mouse Aβ40 ELISA kit (KMB3481; Invitrogen) according to the manufacturer’s instructions. In brief, scramble or PRR-KD MC3T3 cell lysate samples were added to an Aβ40 antibody-coated 96-well plate for 2 h at room temperature. After being washed 4 times with wash buffer, Aβ40 detection antibodies were added to each well for 1h at room temperature and then washed 4 times. Stabilized chromogens were added into each well and incubated in the dark for 30 min. After adding the stop solution, their absorbance at 450 nm was measured using a Synergy Biotek Instrument. Aβ40 levels were calculated based on a calibration curve with standard samples.

### 2.8. Immunostaining

Cells were fixed with fixing solution (4% paraformaldehyde (PFA)/4% sucrose in 1XPBS) (pH ~7.4) for 20 min at room temperature, permeabilized with 0.2% Triton X-100 for 10 min and blocked in 2% bovine serum albumin (BSA) for 1 h in PBS. Subsequently, the cells were incubated with primary antibodies overnight at 4 °C. After being washed 5 times with 0.1% Tween-20 in PBS, cells were incubated with Alexa Fluor-conjugated secondary antibodies (Invitrogen or Jackson ImmunoResearch) for 1–2 h and then washed 5 times. Nuclei were counterstained using DAPI (0.1 µg/mL, Roche) for 5 min and then washed 5 times. Coverslips were mounted onto glass slides with PVA-DABCO cover-slipping solution. Images were captured using confocal microscopy.

### 2.9. Live Cell Imaging and Drug Treatment

All time-lapse images were acquired using a Zeiss LSM 800 (Carl Zeiss, Oberkochen, Germany) with an incubation system and CO_2_ and temperature control. NLT cells or cortical neurons were plated on 35 mm glass-bottom Microwell dishes with 2 mL of the corresponding medium. The glass-bottom dishes were then fitted into a temperature-controlled chamber on the microscope stage for observation at 37 °C under a 5% CO_2_ air atmosphere. Images of regions of interest were taken every 1 min and recorded at the indicated time with 63× objectives.

For the CQ/BafA1 treatment experiment, 2 µL of the drug was added to 2 mL of medium (1:1000) during recordings and was maintained for the indicated time. The following treatments were used: 100 mM chloroquine (vehicle: water, Sigma) and 200 µM bafilomycin A1 (vehicle: DMSO, Sigma).

For the BACE1 inhibitor treatment experiment, six hours after transfection, the culture medium was removed and replaced with 10% FBS DMEM containing 1 µM LY2886721 (vehicle: DMSO, abcam). After 48 h, the cells were subjected to live cell imaging analysis.

### 2.10. In Utero Electroporation

In utero electroporation was performed as described previously [37,45,46]. Briefly, pregnant mice at E15.5 were anesthetized and maintained with 2% isoflurane inhalation and subjected to an abdominal incision to expose the uterus. Expression plasmids (2 μg/μL) plus 0.01% Fast Green (Sigma-Aldrich) were injected into the lateral ventricles of the embryonic brain with a glass capillary. For electroporation, 5 × 50 ms, 36 V square pulses separated by 950 ms intervals were delivered with forceps-type electrodes connected to an ECM 830 electroporator (BTX). The uterus was then carefully repositioned into the abdominal cavity and the abdominal wall and skin were sutured using a surgical needle. The pregnant mouse was warmed in an incubator until it regained consciousness and the pups were reared to the indicated postnatal stages. Under deep anesthesia, the pups were perfused transcardially with phosphate-buffered saline (PBS) followed by 4% paraformaldehyde (PFA) (pH ~7.4). At each time-point, at least six pups (three for each construct mix) were used for data analysis in each set of experiments. The brains of the pups were cut into 80 µm floating slices immediately after perfusion using a vibratome (Leica, VT1000s) cutting system. The slices were subjected to confocal microscopy.

### 2.11. Statistical Analysis

Immunostained cells were imaged under a Zeiss LSM 800 confocal microscope with Zen software. Cells from the control and experimental groups were directly compared and imaged with the same acquisition parameters. For fluorescent quantification, intensity analysis and morphometric measurements of images were performed using ImageJ software. Statistical analysis was performed using OriginPro8.0 (OriginLab) and GraphPad Prism7 (GraphPad) software and the unpaired 2-tailed Student’s *t*-test following a test of the normality of the distribution (*p* > 0.05). The data are presented as the mean ± standard error of the mean (SEM). *p* values less than 0.05 were considered significant. Numbers, replicates and tests to determine statistical significance are stated in the text and in the figure legends of individual experiments.

## 3. Results

### 3.1. Generation of BACE1 Fusion Proteins

**Figure 1:**
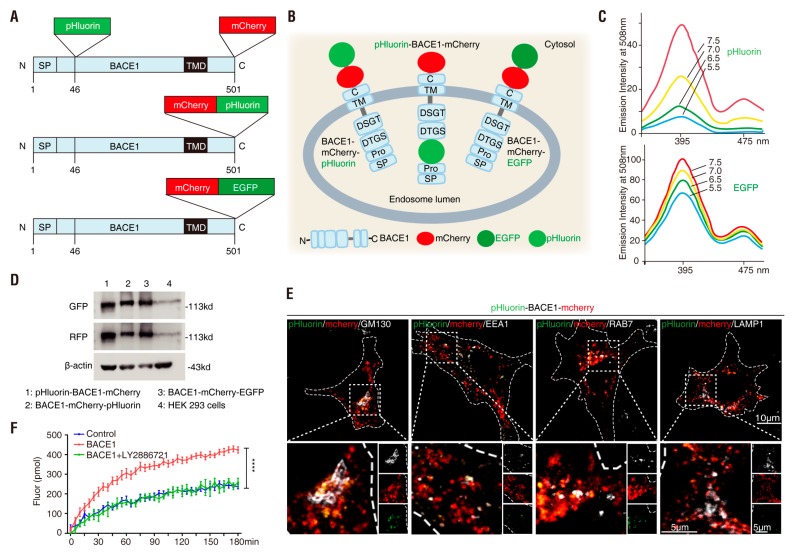
Generation of β-site APP cleaving enzyme 1 (BACE1) fusion proteins (**A**) Schematic of the linear structure of the three BACE1 expression plasmids. N, N terminal; C, C terminal; SP, BACE1 signal peptide; TMD, transmembrane domain; pHluorin and enhanced green fluorescent protein (EGFP), pH-sensitive green fluorescent protein; mCherry, pH-insensitive red fluorescent protein. (**B**) Schematic of the three BACE1 fusion proteins in the endosomal lumen. (**C**) Fluorescence excitation spectra of ecliptic pHluorin and EGFP (adapted from Gero Miesenbock) [47]. (**D**) Western blot analysis of HEK293 cells transfected with the indicated plasmids. (**E**) NLT cells expressing pHluorin-BACE1-mCherry (red and green) and BFP (blue) were immunostained with the subcellular markers: GM130, EEA1, Rab7 and LAMP1 (white). Scale bar, 10 µm or 5 µm. (**F**) BACE1 activity of GFP (Control) and pHluorin-BACE1-mCherry (BACE1) with or without BACE1 inhibitor (LY2886721) in HEK293 cells. Data was shown as mean ± SEM (*n* = 8 from three independent experiments). Significance was calculated with two-way ANOVA with LSD post hoc test; **** *p* < 0.0001

BACE1 consists of an N-terminal signal peptide (SP)(_1–21_), a pro-peptide domain (Pro)(_22–45_) that are removed during BACE1 maturation [13], a catalytic domain (D_93_TGS D_289_SGT)(_46–454_), a transmembrane domain (TMD)(_455–478_) and a C terminus (_479–501_) that is located on the cytosolic side [7,8] (Figure 1A,B). In light of the structure features of BACE1and literature reports that BACE1 activity requires acidic pH [17,18,19], we generated three different fluorescence-tagged BACE1 fusion proteins, named pHluorin-BACE1-mCherry, BACE1-mCherry-pHluorin and BACE1-mCherry-EGFP (Figure 1A,B). In these fusion proteins, pHluorin is a pH sensor that does not emit obvious fluorescence at normal vesicle pH (4.5–6) but emits green fluorescence at basic pH (7–7.5) [47] (Figure 1C); mCherry is a pH-insensitive protein that emits red fluorescence at pH 4.5 to 7.5 and is thus useful to indicate fusion protein expression and localization; and EGFP emits green fluorescence with less sensitivity to pH changes (Figure 1C), which is used as a control for pHluorin. As BACE1 undergoes endocytosis [13], we fused pHluorin with the BACE1 N-terminal (pHluorin-BACE1-mCherry) or C-terminal (BACE1-mCherry-pHlourin) region, which are expected to report pH changes inside or outside of vesicles (e.g., endosomes, late endosomes or lysosomes), respectively (Figure 1B).

We transiently transfected three plasmids into HEK293 cells to test the expression of these fusion proteins. The resulting lysates were subjected to immunoblotting analysis. As expected, ~113 kDa bands (55 kDa-BACE1 + 29 kDa-EGFP/pHluorin + 29 kDa-mCherry) were detected in lysates expressing these plasmids using antibodies specific for GFP or RFP (Figure 1D), suggesting that the three plasmids encoded BACE1 fusion proteins at the right molecular weight. The plasmids were then transfected into NLT cells, which were fixed (using 4% PFA at pH ~7.4) and subjected to immunostaining analysis. Cells expressing pHluorin-BACE1-mCherry showed overlapping green (pHluorin) and red (mCherry) fluorescence, providing additional evidence for in-frame and correct fusion protein expression (Figure 1E). Moreover, the red or green fluorescence partially colocalized with immune-fluorescence signals detected by antibodies specific for GM130 (a marker for the trans-Golgi network), EEA1 (an early endosome marker), Rab7 (a late endosome marker) and LAMP1 (a marker for late endosomes and early lysosomes), exhibiting a subcellular distribution pattern similar to that described for BACE1 in the literature [37,48,49,50,51,52] (Figure 1E). A similar distribution pattern was observed in cells expressing BACE1-mCherry-EGFP or BACE1-mCherry-pHluorin (data not shown). These results demonstrate that fusing pHlourin or mCherry with BACE1 did not alter BACE1′s subcellular distribution, suggesting that the plasmids were constructed successfully.

Furthermore, we examined whether fusing pHluorin or mCherry with BACE1 alters BACE1 activity. HEK293 cells expressing pHluorin-BACE1-mCherry and control plasmid (GFP) were treated with BACE1 inhibitor, LY2886721 or vehicle control for 48 h. Cell lysates were subjected to BACE1 activity assay as described in Methods. Cells expressing pHluorin-BACE1-mCherry showed much higher BACE1 activity than that of controls (Figure 1F), demonstrating its activity. Such an increase of BACE1 activity was abolished in the presence of the BACE1 inhibitor (Figure 1F), suggesting its sensitivity to the inhibitor. Taken together, these results demonstrate that BACE1 fusing with pHlourin or mCherry not only has normal BACE1′s subcellular distribution but also remains its activity.

### 3.2. pHluorin-BACE1-mCherry, a Better pH Sensor for BACE1 in NLT Cells

**Figure 2:**
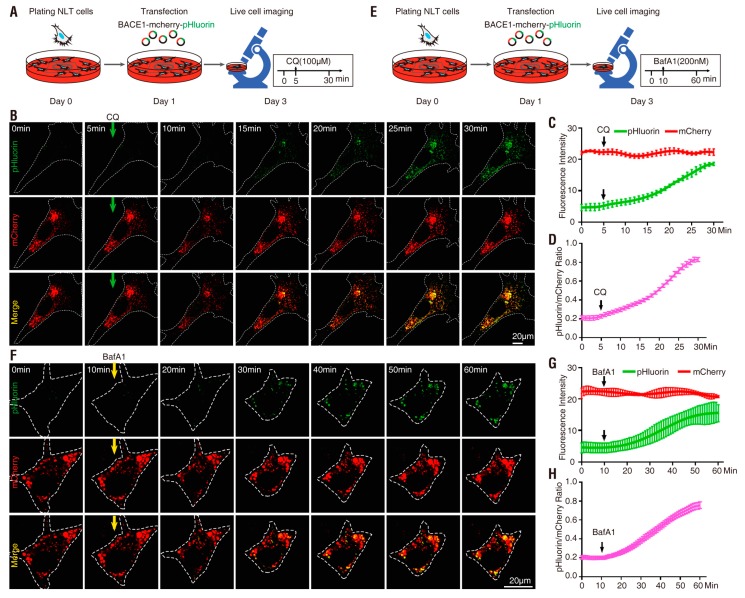
Response of NLT cells expressing BACE1-mCherry-pHluorin to chloroquine (CQ) and bafilomycin A1(BafA1) treatment (**A**) Schematics of live cell imaging experimental procedures. NLT cells were plated on Day 0 and co-transfected with BACE1-mCherry-pHluorin and blue fluorescent protein (BFP) on Day 1. NLT cells expressing BACE1-mCherry-pHluorin were imaged every minute for 30 min on Day 3 and treated with 100 µM CQ after 5 min. (**B**) Representative images from live cell imaging in response to CQ. NLT cells expressing BACE1-mCherry-pHluorin (red and green) were outlined according to BFP expression. Scale bar, 20 µm. (**C**) The fluorescence signal intensity of mCherry (red) and pHluorin (green) in the outlined area was measured using ImageJ and plotted over time in response to CQ. Data were shown as mean ± SEM (*n* > 15 cells from three independent experiments). (**D**) Quantification analysis of the pHluorin/mCherry ratio from (**C**). Data was shown as mean ± SEM (*n* > 15 cells from three independent experiments). (**E**) Schematics of live cell imaging experimental procedures. NLT cells were plated on Day 0 and co-transfected with BACE1-mCherry-pHluorin and BFP on Day 1. NLT cells expressing BACE1-mCherry-pHluorin were imaged every minute for 60 min on Day 3 and treated with 200 nM BafA1 after 10 min. (**F**) Representative images from live cell imaging in response to BafA1. NLT cells expressing BACE1-mCherry-pHluorin (red and green) were outlined according to BFP expression. Scale bar, 20 µm. (**G**) The fluorescence signal intensity of mCherry (red) and pHluorin (green) in the outlined area were measured and plotted over time in response to BafA1. Data were shown as mean ± SEM (*n* > 15 cells from three independent experiments). (**H**) Quantification analysis of the pHluorin/mCherry ratio from (**G**). Data was shown as mean ± SEM (*n* > 15 cells from three independent experiments).

We next examined the pH sensitivity in NLT cells expressing each of these BACE1 fusion proteins by time-lapse imaging analysis. NLT was chosen because it has a spread morphology with relatively large subcellular organelles [50,53]. To detect pH changes in cellular or intracellular compartments, cells transfected with the indicated BACE1 plasmids were treated with chloroquine (CQ) or bafilomycin A1 (BafA1) (Figure 2A,E). CQ is a lysosomotropic agent that concentrates in acidic vesicles and raises their pH, thus inhibiting acidification of these intracellular compartments [54,55]. BafA1 is a macrolide antibiotic that is a selective inhibitor of the vacuolar-type ATPase (V-ATPase), an essential proton pump for maintaining the vesicular pH [56,57]. At nanomolar concentrations, BafA1 disrupts the vesicular proton gradient and ultimately increases the pH of acidic vesicles [58].

NLT cells expressing BACE1-mCherry-pHluorin were first tested as illustrated in Figure 2A. In the absence of CQ (0 min), the mCherry^+^ but not pHluorin fluorescence was detected and mCherry appeared to be distributed in intracellular vesicles, as observed in fixed NLT cells (Figure 1E and Figure 2B). Upon CQ treatment, whereas the mCherry^+^ puncta remained unchanged (in terms of both distribution and intensity), time-dependent increases in pHluorin fluorescence intensity were observed (Figure 2B–D). The pHluorin fluorescence began to increase at ~5 min, was noticeable at ~10 min and peaked at ~25 min following CQ treatment (Figure 2B–D). The basal every minute was ~0.2 (0.211 ± 0.079) and was elevated to ~0.9 (~4.5-fold-increase) by CQ (Figure 2D and Table 1). Similar to CQ treatment, BafA1 also increased the pHluorin but not mCherry fluorescence intensity (Figure 2E–H). However, this change required a longer time (~14 min vs. 5 min) and was less effective (~4.0-fold vs. 4.5-fold) for BafA1 (at a concentration of 200 nM) than for CQ (at a concentration of 100 µM) to increase pHluorin fluorescence (Figure 2 and Table 1).

**Figure 3:**
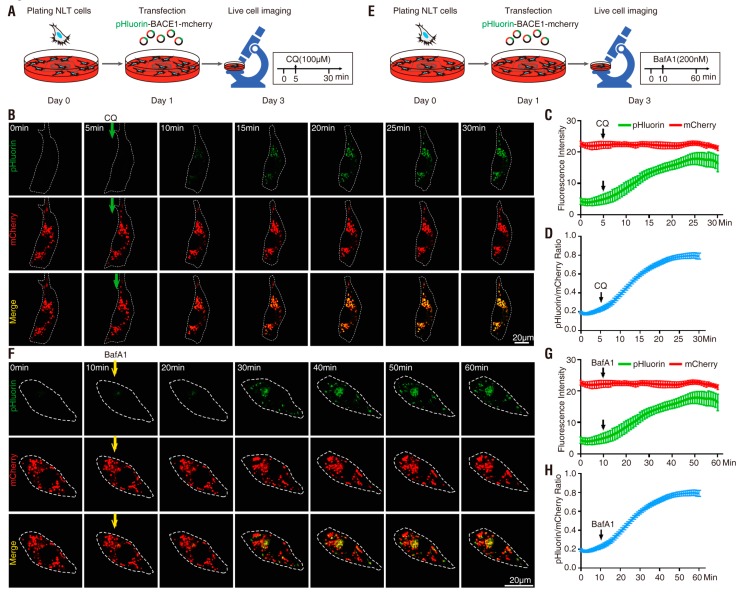
Response of NLT cells expressing pHluorin-BACE1-mCherry to CQ and BafA1 treatment (**A**) Schematics of live cell imaging experimental procedures. NLT cells were plated on Day 0 and co-transfected with pHluorin-BACE1-mCherry and BFP on Day 1. NLT cells expressing BACE1-mCherry-pHluorin were imaged every minute for 30 min on Day 3 and treated with 100 µM CQ after 5 min. (**B**) Representative images from live cell imaging in response to CQ. NLT cells expressing pHluorin-BACE1-mCherry (red and green) were outlined according to BFP expression. Scale bar, 20 µm. (**C**) The fluorescence signal intensity of mCherry (red) and pHluorin (green) in the outlined area was measured using ImageJ and plotted along with time in response to CQ. Data were shown as mean ± SEM (*n* > 15 cells from three independent experiments). (**D**) Quantification analysis of the pHluorin/mCherry ratio from C. Data was shown as mean ± SEM (*n* > 15 cells from three independent experiments). (**E**) Schematics of live cell imaging experimental procedures. NLT cells were plated on Day 0 and co-transfected with pHluorin-BACE1-mCherry and BFP on Day 1. NLT cells expressing pHluorin-BACE1-mCherry were imaged every minute for 60 min on Day 3 and treated with 200 nM BafA1 after 10 min. (**F**) Representative images from live cell imaging in response to BafA1. NLT cells expressing pHluorin-BACE1-mCherry (red and green) were outlined according to BFP expression. Scale bar, 20 µm. (**G**) The fluorescence signal intensity of mCherry (red) and pHluorin (green) in the outlined area was measured and plotted over time in response to BafA1. Data were shown as mean ± SEM (*n* > 15 cells from three independent experiments). (**H**) Quantification analysis of the pHluorin/mCherry ratio from G. Data was shown as mean ± SEM (*n* > 15 cells from three independent experiments).

**Figure 4:**
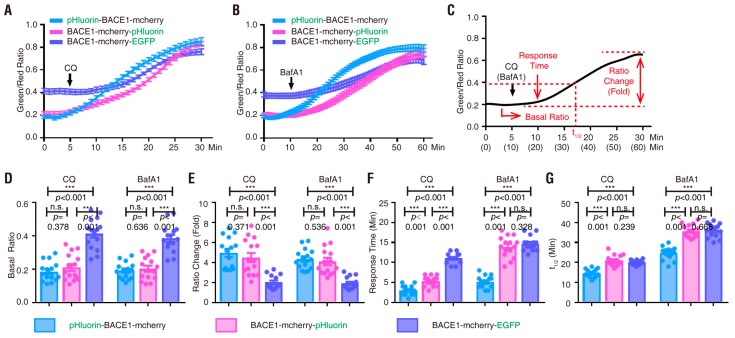
Summary of BACE1 fusion proteins in response to CQ and BafA1 in NLT cells (**A**) Quantification analysis of the green/red ratio of three BACE1 fusion proteins in response to CQ. (**B**) Quantification analysis of the green/red ratio of three BACE1 fusion proteins in response to BafA1. (**C**) Illustration of the term “Basal ratio,” “Ratio change (fold),” “Response time” and “t_1/2_.” (**D**) Quantification analysis of the “Basal ratio” of three BACE1 fusion proteins in response to CQ and BafA1. Significance was calculated with one-way ANOVA with LSD post hoc test; n.s. *p* > 0.05, *** *p* < 0.001. (**E**) Quantification analysis of the “Ratio change (fold)” of three BACE1 fusion proteins in response to CQ and BafA1. Significance was calculated with one-way ANOVA with LSD post hoc test; n.s. *p* > 0.05, *** *p* < 0.001. (**F**) Quantification analysis of the “Response time” of three BACE1 fusion proteins in response to CQ and BafA1. Significance was calculated with one-way ANOVA with LSD post hoc test; n.s. *p* > 0.05, *** *p* < 0.001. (**G**) Quantification analysis of the “t_1/2_” of three BACE1 fusion proteins in response to CQ and BafA1. Significance was calculated with one-way ANOVA with LSD post hoc test; n.s. *p* > 0.05, *** *p* < 0.001.

Note that after BACE1 endocytosis, the C-terminal-fused pHlourin (in BACE1-mCherry-pHluorin) may face the cytosol, not the intracellular vesicle lumen, where it is more acidic (Figure 1B) [7,8]. We thus next characterized pHluorin-BACE1-mCherry, in which the pHluorin is fused to the N-terminus of BACE1 and points to the lumen side of the vesicles (Figure 1B). NLT cells expressing this BACE1 fusion protein were examined as illustrated in Figure 3A,E. Indeed, cells expressing this N-terminal pHluorin fused BACE1 (pHluorin-BACE1-mCherry) showed a quicker response to CQ (~3.1 min vs. ~5.3 min) and BafA1 (~5.2 min vs. ~14 min) than the cells expressing BACE1-mCherry-pHluorin (Figure 3 and Figure 4 and Table 1). In addition, the t_1/2_ (the time needed to reach 50% of the maximal response to CQ/BafA1) was also faster in pHluorin-BACE1-mCherry-expressing cells than in BACE1-mCherry-pHluorin-expressing cells (Figure 4G). However, the pHluorin/mCherry ratio changes induced by both CQ and BafA1 were comparable between pHluorin-BACE1-mCherry- and BACE1-mCherry-pHluorin-expressing cells (Figure 4E, Table 1). These results suggest that pHluorin facing the cytosol or the vesicle lumen could report pH changes induced by CQ/BafA1 but the pHluorin facing the vesicle lumen (pHluorin-BACE1-mCherry) responded to the pH changes faster than the pHluorin facing the cytosol (BACE1-mCherry-pHluorin) (Figure 4 and Table 1).

Finally, we examined NLT cells expressing BACE1-mCherry-EGFP in response to CQ-induced pH changes. In comparison with BACE1-mCherry-pHluorin or pHluorin-BACE1-mCherry, although EGFP also responded to CQ treatment, its response was much slower (11 min vs. 3–5 min), with a smaller fold increase in its fluorescence intensity (2-fold vs. 5-fold), than that of pHluorin (Figure S1, Figure 4 and Table 1). Additionally, the basal green fluorescence and the ratio of green/red fluorescence (without CQ) in cells expressing BACE1-mCherry-EGFP were much higher than those of cells expressing BACE1-mCherry-pHluorin or pHluorin-BACE1-mCherry (~0.4 vs. ~0.2) (Figure S1, Figure 4 and Table 1). Taken together, these results suggest that pHluorin-BACE1-mCherry is a better pH sensor for BACE1 in NLT cells.

We also examined whether the BACE1 inhibitor—LY2886721—suppresses BACE1 activity through vesicular pH. NLT cells expressing pHluorin-BACE1-mCherry were treated with LY2886721 or vehicle control for 48 h and subjected to pHluorin fluorescence imaging analysis. The pHluorin fluorescence was slightly increased but without significant difference (Figure S2). These results suggest that LY2886721 inhibition of BACE1 may through a mechanism independent on vesicular pH.

### 3.3. pHluorin-BACE1-mCherry, a Better pH Sensor for BACE1 in Cultured Neurons

**Figure 5:**
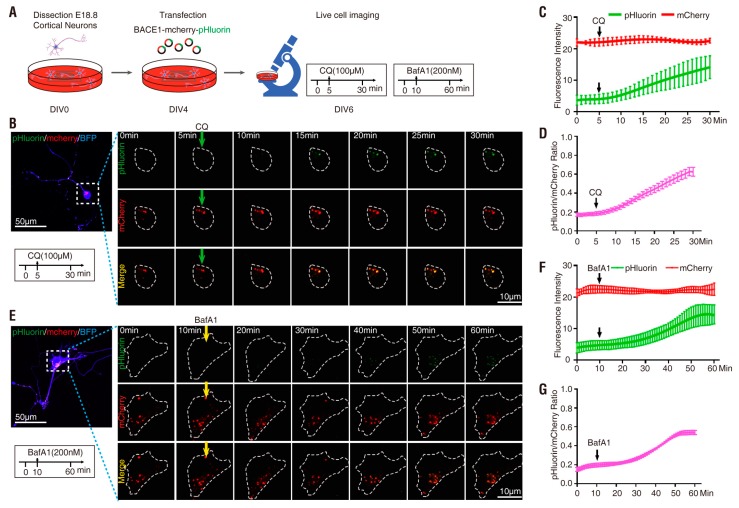
Response of primary cortical neurons expressing BACE1-mCherry-pHluorin to CQ and BafA1 treatment (**A**) Schematics of live cell imaging experimental procedures. Cortical neurons were dissected from E18.5 mice and plated on 35 mm glass-bottom culture dishes at DIV0. Neurons were co-transfected with BACE1-mCherry-pHluorin and BFP at DIV4 and imaged every minute for 30 min at DIV6 and treated with 100 µM CQ after 5 min or imaged every minute for 60 min at DIV6 and treated with 200 nM BafA1 after 10 min. (**B**) Representative images from live cell imaging in response to CQ. The soma expressing BACE1-mCherry-pHluorin (red and green) were outlined according to BFP expression. Scale bar, 10 µm or 50 µm. (**C**) The fluorescence signal intensity of mCherry (red) and pHluorin (green) in the outlined area was measured using ImageJ and plotted over time in response to CQ. Data were shown as mean ± SEM (*n* > 15 neurons from three independent experiments). (**D**) Quantification analysis of the pHluorin/mCherry ratio from C. Data was shown as mean ± SEM (*n* > 15 neurons from three independent experiments). (**E**) Representative images from live cell imaging in response to BafA1. The soma expressing BACE1-mCherry-pHluorin (red and green) were outlined according to BFP expression. Scale bar, 10 µm or 50 µm. (**F**) The fluorescence signal intensity of mCherry (red) and pHluorin (green) in the outlined area was measured and plotted over time in response to BafA1. Data were shown as mean ± SEM (*n* > 15 neurons from three independent experiments). (**G**) Quantification analysis of the pHluorin/mCherry ratio from F. Data was shown as mean ± SEM (*n* > 15 neurons from three independent experiments).

**Figure 6:**
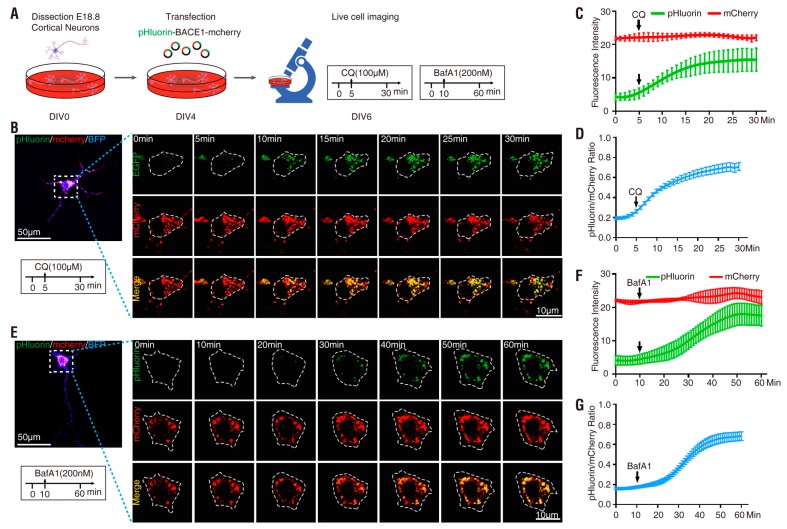
Response of primary cortical neurons expressing pHluorin-BACE1-mCherry to CQ and BafA1 treatment (**A**) Schematics of live cell imaging experimental procedures. Cortical neurons were dissected from E18.5 mice and plated on 35 mm glass-bottom culture dishes at DIV0. Neurons were co-transfected with pHluorin-BACE1-mCherry and BFP at DIV4 and imaged every minute for 30 min at DIV6 and treated with 100 µM CQ after 5 min or imaged every minute for 60 min at DIV6 and treated with 200 nM BafA1 after 10 min. (**B**) Representative images from live cell imaging in response to CQ. The soma expressing pHluorin-BACE1-mCherry (red and green) were outlined according to BFP expression. Scale bar, 10 µm or 50 µm. (**C**) The fluorescence signal intensity of mCherry (red) and pHluorin (green) in the outlined area was measured using ImageJ and plotted over time in response to CQ. Data were shown as mean ± SEM (*n* > 15 neurons from three independent experiments). (**D**) Quantification analysis of the pHluorin/mCherry ratio from C. Data was shown as mean ± SEM (*n* > 15 neurons from three independent experiments). (**E**) Representative images from live cell imaging in response to BafA1. The soma expressing pHluorin-BACE1-mCherry (red and green) were outlined according to BFP expression. Scale bar, 10 µm or 50 µm. (**F**) The fluorescence signal intensity of mCherry (red) and pHluorin (green) in the outlined area was measured and plotted over time in response to BafA1. Data were shown as mean ± SEM (*n* > 15 neurons from three independent experiments). (**G**) Quantification analysis of the pHluorin/mCherry ratio from F. Data was shown as mean ± SEM (*n* > 15 neurons from three independent experiments).

**Figure 7:**
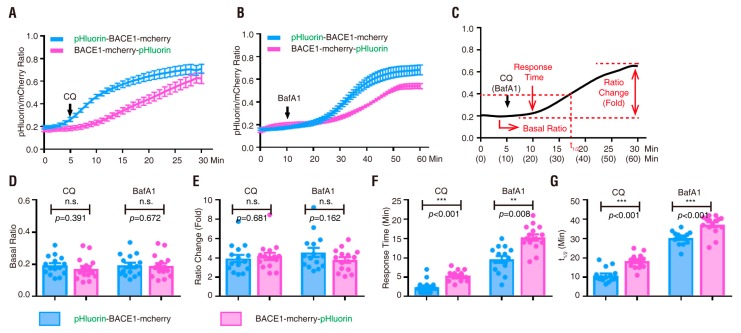
Summary of BACE1 fusion proteins in response to CQ and BafA1 in primary cortical neurons (**A**) Quantification analysis of the pHluorin/mCherry ratio of two BACE1 fusion proteins in response to CQ. (**B**) Quantification analysis of the pHluorin/mCherry ratio of two BACE1 fusion proteins in response to BafA1. (**C**) Illustration of the term “Basal ratio,” “Ratio change (fold),” “Response time” and “t_1/2_.” (**D**) Quantification analysis of the “Basal ratio” of three BACE1 fusion proteins in response to CQ and BafA1. Significance was calculated with Student’s *t*-test; n.s. *p* > 0.05. (**E**) Quantification analysis of the “Ratio change (fold)” of three BACE1 fusion proteins in response to CQ and BafA1. Significance was calculated with Student’s *t*-test; n.s. *p* > 0.05. (**F**) Quantification analysis of the “Response time” of three BACE1 fusion proteins in response to CQ and BafA1. Significance was calculated with Student’s *t*-test; *** *p* < 0.001, ** *p* < 0.01. (**G**) Quantification analysis of the “t_1/2_” of three BACE1 fusion proteins in response to CQ and BafA1. Significance was calculated with Student’s *t*-test; *** *p* < 0.001.

We further investigated whether pHluorin is more sensitive to pH changes when it faces the vesicle side than when it faces the cytosol side in primary cortical neurons. Primary cortical neurons (E18.5, DIV 4) were transfected with pHluorin-BACE1-mCherry or BACE1-mCherry-pHluorin and subjected to time-lapse imaging analyses with CQ or BafA1 treatments as illustrated in Figure 5A,F and Figure 6A,F. Again, the basal ratios of pHluorin/mCherry and CQ/BafA1-increased ratios of pHluorin/mCherry in neurons expressing pHluorin-BACE1-mCherry were comparable to those of neurons expressing BACE1-mCherry-pHluorin (Figure 5, Figure 6, Figure 7D,E and Table 2). However, the response times to CQ and BafA1 were much faster in neurons expressing pHluorin-BACE1-mCherry than in neurons expressing BACE1-mCherry-pHluorin (~2.4 min vs. ~5.3 min to CQ; ~9.6 min vs. ~15.3 min to BafA1) (Figure 7F, Table 2). The t_1/2_ was also faster in pHluorin-BACE1-mCherry-expressing neurons than in BACE1-mCherry-pHluorin neurons (~11 min vs. ~18.3 min to CQ; ~30 min vs. ~37.2 min to BafA1) (Figure 7G, Table 2). These results, in line with the data from NLT cells, provide additional evidence that pHluorin-BACE1-mCherry is a better pH sensor for BACE1.

### 3.4. PRR Regulation of phluorin-BACE1-mCherry and BACE1 Activity in Culture and In Vivo

**Figure 8:**
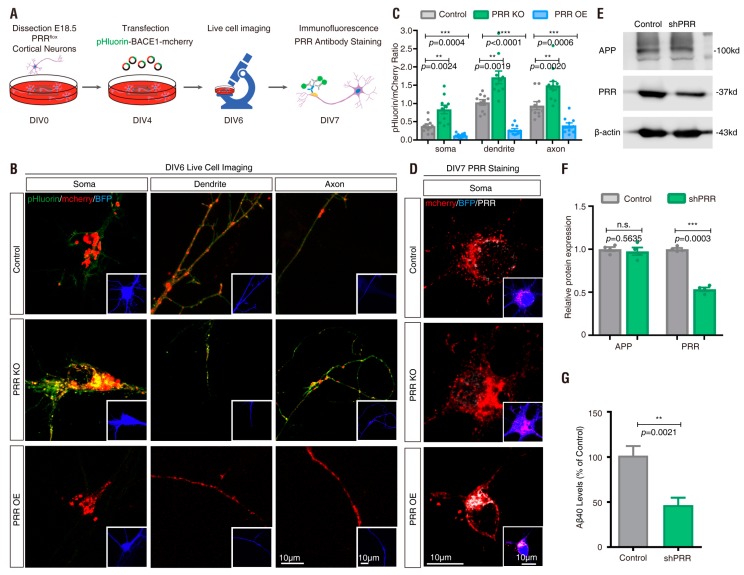
Regulation of pHluorin-BACE1-mCherry and BACE1 activity by (pro)renin receptor (PRR) in vitro (**A**) Schematics of live cell imaging experimental procedures. Cortical neurons were dissected from E18.5 PRR^f/f^ mice and plated on 35 mm glass-bottom culture dishes at DIV0. Neurons were co-transfected with pHluorin-BACE1-mCherry and BFP without Cre (control) or with Cre (PRR KO) or with PRR (PRR OE) at DIV4 and underwent live cell imaging at DIV6. Immunostaining of PRR was performed at DIV7. (**B**) Confocal live cell imaging of transfected neurons at DIV6 was carried out and representative images are shown. Scale bar, 10 µm. (**C**) Quantification analysis of the pHluorin/mCherry ratio from (**B**). Data was shown as mean ± SEM (*n* > 15 neurons from three independent experiments). Significance was calculated with Student’s *t*-test; **** *p* < 0.0001, *** *p* < 0.001, ** *p* < 0.01. (**D**) Immunostaining of PRR in transfected neurons at DIV7 was carried out and representative images are shown. Scale bar, 10 µm. (**E**) Western blot analysis of MC3T3 cells infected with control lentivirus or shRNA-PRR lentivirus. (**F**) Quantification analysis of relative protein expression from (**E**). Data was shown as mean ± SEM (*n* = 4 from three independent experiments). Significance was calculated with Student’s *t*-test; n.s. *p* > 0.05, *** *p* < 0.001. (**G**) Aβ40 levels of control or PRR-KD MC3T3 cells. Data was shown as mean ± SEM (*n* = 6 from three independent experiments). Significance was calculated with Student’s *t*-test; ** *p* < 0.01.

PRR [(pro)renin receptor]—also identified as adenosine triphosphatase (ATPase), H+-transporting, lysosomal accessory protein 2 (ATP6AP2)—is a subunit of the V-ATPase [59,60,61,62]. The V-ATPase resides within many intracellular compartments such as endosomes, lysosomes and secretory vesicles, which are crucial for maintaining the intracellular vesicular acidic environment [63]. PRR ablation in cultured cells results in the downregulation of several subunits of the V0 complex of the V-ATPase and impairs vesicle acidification [44,62,64,65]. BACE1 activity is optimal in acidic environments [17,18,19]. In light of these observations, we wondered whether BACE1 activity is regulated by PRR. To further investigate pHluorin-BACE1-mCherry’s sensitivity to the V-ATPase-driven vesicle pH, we cultured primary cortical neurons from E18.5 floxed PRR (PRR^f/f^) mice and transfected pHluorin-BACE1-mCherry with or without a plasmid encoding Cre into the cultured neurons at DIV4 (Figure 8A). As shown in Figure 8D, Cre-expressing neurons showed little PRR/ATP6AP2 immunostaining, suggesting that PRR knock-out (KO) occurred in these neurons. The transfected neurons (at DIV 6) were subjected to live cell time-lapse imaging analysis. As expected, a much higher ratio of pHluorin/mCherry was detected in PRR-KO neurons than in control neurons (Figure 8B,C), suggesting that inhibition of the V-ATPase by PRR-KO, similar to BafA1, impairs vesicular pH and increases pHluorin fluorescence intensity.

We next asked whether overexpression of PRR in pHluorin-BACE1-mCherry^+^ neurons affects pHluorin fluorescence. The primary cortical neurons at DIV4 were co-transfected the plasmid of PRR with pHluorin-BACE1-mCherry. These neurons were subjected to live cell time-lapse imaging analyses at DIV 6 and subjected to immunostaining at DIV 7 (Figure 8A). Immunostaining analysis using an antibody specific for PRR/ATP6AP2 verified PRR overexpression (Figure 8D). Time-lapse imaging analysis showed that the ratio of pHluorin/mCherry in PRR overexpressing neurons was lower than that in WT control neurons (Figure 8B,C), in contrast to the PRR-KO. These results not only verified pHluorin-BACE1-mCherry as a pH sensor for BACE1 but also implicated PRR/V-ATPase as a potential regulator of BACE1 activity.

We then examined BACE1 activity between control and PRR-KD (knockdown) cells. PRR was knocked down by infection of MC3T3 cells (an osteoblastic cell line that expresses PRR, APP and BACE1) with shRNA-PRR lentivirus [44,66] (Figure 8E). Aβ (1–40) levels were measured by ELISA and used to represent BACE1 activity. The Aβ levels were lower in PRR-KD cells (Figure 8F) than in the control groups, supporting the view that the PRR/vesicle acidic environment is a positive regulator of BACE1 activation.

**Figure 9:**
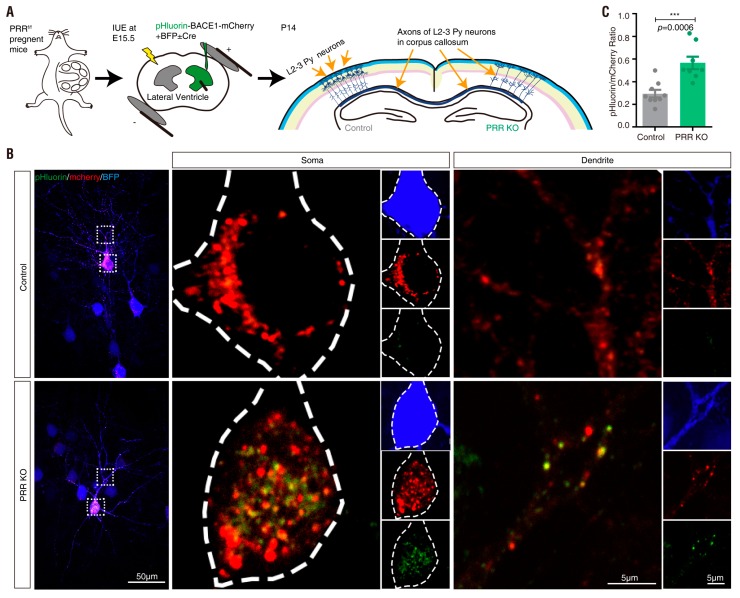
PRR regulation of pHluorin-BACE1-mCherry and BACE1 activity in vivo (**A**) Schematics of in utero electroporation experimental procedures. PRR^f/f^ embryos were in utero electroporated with plasmids of pHluorin-BACE1-mCherry and BFP (control) or pHluorin-BACE1-mCherry, BFP and Cre (PRR KO) at E15.5. The neocortical brain sections were collected at P14. (**B**) Representative Z-stack projection images from neocortical brain sections was shown. Scale bar, 50 µm/5 µm. (**C**) Quantification analysis of the pHluorin/mCherry ratio from (**B**). Data was shown as mean ± SEM (*n* > 30 neurons from three independent experiments. Significance was calculated with Student’s *t*-test; *** *p* < 0.001.

To address whether PRR-KO affects the distribution of active BACE1 in vivo, we took advantage of the in utero electroporation (IUE) assay and the PRR^f/f^ embryos for the following reasons. First, IUE is a quick cell based in vivo method for functional analysis of an interest gene. Second, IUE is cost effective, without large amount of animal breeding. Third, it can be used to co-express or suppress multiple genes simultaneously in the same neurons, which is not doable by use of recombinant viral systems. The plasmids encoding pHluorin-BACE1-mCherry and Cre/control were co-electroporated into the NSCs/NPCs in the ventricular zone of PRR^f/f^ embryos at E15.5. After IUE, neonatal cortical brain samples at postnatal (P) 14 were collected (Figure 9A). Expression of the Cre plasmid (PRR-KO) increased the ratio of pHluorin/mCherry compared with that in the control neurons (Figure 9B,C). These results revealed the potential of pHluorin-BACE1-mCherry as a useful marker for active BACE1 in vivo.

### 3.5. APPswe Regulation of pHluorin-BACE1-mCherry in Culture and In Vivo

BACE1-mediated cleavage of APP results in the release of a soluble amino-terminal fragment termed sAPPβ. Mutations in the APP gene linked to familial forms of AD are found very close to the BACE1 cleavage site and generally increase Aβ generation. The Swedish double mutation, which was found in an individual member of a Swedish family, is located directly N-terminally to the β-secretase cleavage site and allows much more efficient β-secretase cleavage of APP and turnover to Aβ. To further address pHluorin-BACE1-mCherry’s sensitivity to the Swedish mutation of APP, we first co-transfected pHluorin-BACE1-mCherry with or without Flag-APP in NLT cells (Figure S3A). We found that the ratio of pHluorin/mCherry in APP overexpressing NLT cells was significantly decreased compared to that in the control group (Figure S3B,C). Immunostaining analysis using an antibody against sAPPβ (to label the BACE1 cleavage product of APP) also showed increased sAPPβ levels on Day 4 in APP-overexpressing NLT cells (Figure S3D).

**Figure 10:**
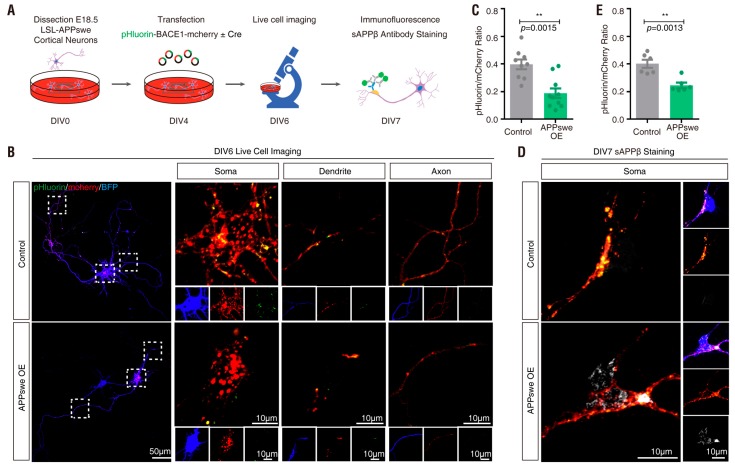
Regulation of pHluorin-BACE1-mCherry and BACE1 activity by Swedish mutant amyloid precursor protein (APPswe) in vitro (**A**) Schematics of live cell imaging experimental procedures. Cortical neurons were dissected from E18.5 LSL-APPswe mice and plated on 35 mm glass-bottom culture dishes at DIV0. Neurons were co-transfected with pHluorin-BACE1-mCherry and BFP without Cre (control) or with Cre (APPswe OE) at DIV4 and underwent live cell imaging at DIV6. Immunostaining of sAPPβ was performed at DIV7. (**B**) Confocal live cell imaging of transfected neurons at DIV6 was carried out and representative images are shown. Scale bar, 10 µm. (**C**) Quantification analysis of the pHluorin/mCherry ratio from (**B**). Data was shown as mean ± SEM (*n* > 10 neurons from three independent experiments). Significance was calculated with Student’s *t*-test; ** *p* < 0.01. (**D**) Immunostaining of sAPPβ in transfected neurons at DIV7 was carried out and representative images are shown. Scale bar, 10 µm. (**E**) Quantification analysis of the pHluorin/mCherry ratio from (**D**). Data was shown as mean ± SEM (*n* > 10 neurons from three independent experiments). Significance was calculated with Student’s *t*-test; ** *p* < 0.01.

We then examined whether APPswe overexpression can influence the pHluorin fluorescence intensity in cortical neurons. We cultured primary cortical neurons from E18.5 LSL-APPswe mice and transfected pHluorin-BACE1-mCherry with or without a plasmid encoding Cre into the cultured neurons at DIV4 (Figure 10A). Cre-expressing neurons showed strong sAPPβ immunostaining signal (Figure 10D), suggesting APPswe overexpression (OE) in these neurons. The transfected neurons (at DIV 6) were subjected to live cell time-lapse imaging analysis. As expected, a much lower ratio of pHluorin/mCherry was detected in APPswe-OE neurons than in control neurons (Figure 10B,C), suggesting that activation of BACE1 by APPswe overexpression, as observed for PRR overexpression, decreases pHluorin fluorescence intensity.

**Figure 11:**
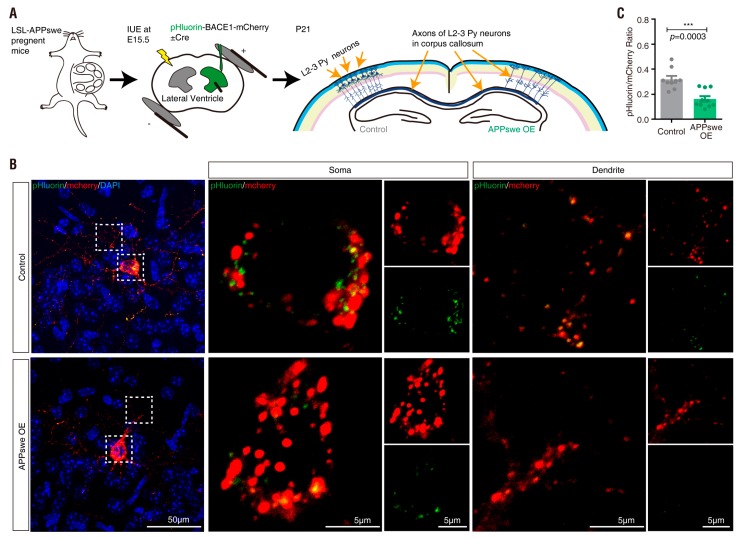
APPswe regulation of pHluorin-BACE1-mCherry and BACE1 activity in vivo (**A**) Schematics of in utero electroporation experimental procedures. LSL-APPswe embryos were in utero electroporated with plasmids of pHluorin-BACE1-mCherry and BFP (control) or pHluorin-BACE1-mCherry and Cre (APPswe OE) at E15.5. The neocortical brain sections were collected at P21. (**B**) Representative Z-stack projection images from neocortical brain sections was shown. Scale bar, 50 µm/5 µm. (**C**) Quantification analysis of the pHluorin/mCherry ratio from (**B**). Data was shown as mean ± SEM (*n* > 30 neurons from three independent experiments). Significance was calculated with Student’s *t*-test; *** *p* < 0.001.

Finally, to address whether APPswe overexpression affects the distribution of active BACE1 in vivo, we took advantage of the in utero electroporation (IUE) assay and the LSL-APPswe embryos. The plasmids encoding pHluorin-BACE1-mCherry and Cre/control were co-electroporated into the NSCs/NPCs in the ventricular zone of LSL-APPswe embryos at E15.5. After IUE, neonatal cortical brain samples at postnatal (P) 21 were collected (Figure 11A). Expression of the Cre plasmid (APPswe-OE) decreased the ratio of pHluorin/mCherry compared with that of control neurons (Figure 11B,C). These results revealed the potential of pHluorin-BACE1-mCherry as a useful marker for active BACE1 in vivo.

## 4. Discussion

Here we present evidence for pHluorin-BACE1-mCherry as a BACE1 activity reporter in cultured cells and in vivo. This BACE1 reporter is sensitive to intracellular vesicular pH changes and the ratio of pHluorin/mCherry fluorescence in cultured cells correlates with BACE1 activity. Interestingly, using this reporter and/or biochemical assays to measure BACE1 activity, we found that both PRR and APPswe are crucial regulators of BACE1 activity. Thus, these studies demonstrate that this BACE1 reporter might be a useful tool not only for the identification of molecular regulators of BACE1 but also for screening BACE1 inhibitors and drug development.

pHluorin-BACE1-mCherry was designed based on the following observations. First, pHluorin (a GFP mutant) has been used as a powerful tool to monitor pH changes of individual cellular organelles and compartments in mammalian cells because it is sensitive to changes in the acidity of its environment in the physiological range [47,67,68]. Second, various pH levels exist in different subcellular organelles. For example, the pH of the cytosol is ~7.2, the pH of the endoplasmic reticulum (ER) is ~7.2, the pH of the trans-Golgi network from cis (~6.7) to trans (~6) gradually decreases, the pH of secretory vesicles is ~5.5, the pH of recycling endosome is ~6.5, the pH of early endosomes is ~6.3, the pH of late endosomes is ~5.5 and the pH of lysosomes is ~4.7 [68,69,70]. Third, genetically fusing GFP or pHluorin with a specific membrane protein has been proven to be successful in sensing pH changes in the lumen of different vesicles [71,72,73,74,75,76,77]. Many studies have exploited the potential of GFP and its mutations to sense the pH dynamics of different intracellular organelles, such as the cytoplasm [78], peroxisomes [71], the trans-Golgi network, endosomes [72] and lysosomes [73]. There are also studies using pHluorin as a dynamic marker of endocytosis [74,75], a marker of protein surface expression [79,80,81] or a marker of autophagy [76,77]. Fourth, BACE1 activity requires an acidic environment [17,18,19]. In light of these observations, we fused BACE1 to the acid-stable fluorescent protein mCherry and acid–unstable fluorescent protein pHluorin or EGFP and generated three different BACE1 fusion proteins named pHluorin-BACE1-mCherry, BACE1-mCherry-pHluorin and BACE1-mCherry-EGFP (Figure 1). The double tag in these fusion proteins can be used for live cell imaging analysis, which may reveal information about the dynamic active/inactive state of BACE1.

By comparison of the three BACE1 fusion proteins, we found that the N-terminal-fused pHluorin is more sensitive to pH changes in response to CQ/BafA1 treatment. Our results are in line with reports that pHluorin is most sensitive to acidic pH [47,77]. Our results also suggest that the BACE1 N-terminal-fused pHluorin (which faces the vesicle lumen side) responds to pH changes more quickly than the C-terminal-fused pHluorin (which faces the cytosol). However, the exact mechanisms for the changes of the pHluorin fluorescence intensity in BACE1-mCherry-pHluorin expressing cells in response to CQ/BafA1 remain unclear. We speculate that CQ/BafA1 at the concentrations of 100 mM/200 µM may not only affect the pH inside the vesicles but also change the local cytosol environment. At lower concentrations, CQ/BafA1 may be more specific, only affecting the vesicular pH, without “side effect.” But this speculation requires additional evidence.

Note that pHluorin-BACE1 has been reported to visualize the surface trafficking of BACE1 in live cells [82] and that BBS-BACE1-YFP, in which BBS is a short peptide from the α-bungarotoxin binding site that can bind fluorescently labeled α-bungarotoxin in live transfected cells, has been used to visualize intracellular/endocytosed BACE1 [83]. Our plasmid, pHluorin-BACE1-mCherry, has the advantages of both pHluorin-BACE1 and BBS-BACE1-YFP. Also notice a report by Utpal Das et al. [84], who use a bimolecular fluorescence complementation (BiFC) method to directly visualize APP and BACE1 interaction. Each method has its advantage and disadvantages. Whereas this BiFC method provides concrete insights into APP-BACE1 interaction and the amyloidogenic pathway, our BACE1 fusion plasmid may reveal useful information regarding active BACE1′s subcellular distribution in culture and in vivo. But, our BACE1 fusion plasmid may not necessarily report BACE1 activity towards Aβ generation. Thus, a combination of both experiments may be necessary for our understanding of Aβ production.

pHluorin-BACE1-mCherry appears to be a useful tool for identifying BACE1 regulators. As described above and reported in the literature, acidic environments affect BACE1 activity [17,18,19] and the acidification of subcellular compartments along the secretory and endocytic pathways depends on the V-ATPase [63,69,85,86]. PRR [(pro)renin receptor], also called ATP6AP2, is a critical regulator of the V-ATPase [59,60,61,62]. Interestingly, PRR loss is believed to be a risk factor for the pathogenesis of Parkinsonism [87,88,89]. Inhibition of the V-ATPase by BafA1 or PRR-KO increases but overexpression of PRR decreases pHluorin fluorescence intensity (Figure 3F, Figure 6G, Figure 8B, Figure 9B). In addition, Aβ level was lower in PRR-KD MC3T3 cells (Figure 8F). These results reconfirmed the requirement of the acidic environment for BACE1 activation and implicated PRR as a novel regulator of BACE1 activity.

In addition to PRR, we found that APPswe acts not only as a substrate of BACE1 [8,90,91,92,93] but also as a potential regulator/activator of BACE1. Overexpression of APPswe resulted in a reduction in the pHluorin/mCherry fluorescence ratio in pHluorin-BACE1-mCherry-expressing cells (Figure S3 and Figure 10) and in vivo (Figure 11). These results implicate that APPswe may affect the vesicular pH and thus increases BACE1 activation. However, this view has no literature support and requires additional evidence and this view does not exclude the current view that the increased availability of APPswe substrates may lead to the increased APPswe cleavage by BACE1. Both views are not mutually exclusive and likely to be involved in the increase of Aβ production. If this view is true, it raises another question as to whether other BACE1 substrates play a regulatory role in BACE1 activation. Several BACE1 substrates have been identified in addition to APP, which include Golgi-localized membrane-bound α2,6-sialyltransferase [94], P-selectin glycoprotein ligand-1 (PSLG-1) [95], the APP homolog proteins APLP1 and APLP2 [96,97,98], low-density lipoprotein receptor-related protein (LRP) [99], voltage-gated sodium channel (Nav1) β2 subunit (Navβ2) [100,101], neuregulin-1 (NRG1) [102,103] and neuregulin-3 (NRG3) [104]. By using our new tool (pHluorin-BACE1-mCherry) as an active BACE1 reporter, we hope to address this question in future studies.

pHluorin-BACE1-mCherry may also be useful in screening BACE1 inhibitors and identifying potential BACE1 substrates. BACE1 is a prime therapeutic target for lowering cerebral Aβ concentrations in Alzheimer’s disease and the clinical development of BACE1 inhibitors is being intensely investigated. Using our new tool, pHluorin-BACE1-mCherry, as an active BACE1 reporter, the dynamic BACE1 activity in vitro or in vivo can be visualized to screen the bioavailability of BACE1 inhibitors instead of measuring BACE1 activity in cultured neurons or CSF (including Aβ40, Aβ42 and sAPPβ). In summary, it is our hope that pHluorin-BACE1-mCherry may serve as a reporter useful not only for the identification of molecular regulators of BACE1 but also for the screen of BACE1 inhibitors and drug development.

## Figures and Tables

**Table 1:** Summary of BACE1 plasmids in response to CQ and BafA1 in NLT cells.

Plasmid	Drug Treatment	Basal Ratio	Ratio Change (fold)	Response Time (min)	t_1/2_ (min)
pHluorin-BACE1-mCherry	CQ	0.185 ± 0.059	4.971 ± 1.548	3.067 ± 1.163	14.673 ± 2.038
BACE1-mCherry-pHluorin	CQ	0.211 ± 0.079	4.505 ± 1.774	5.333 ± 1.234	20.960 ± 2.681
BACE1-mCherry-EGFP	CQ	0.414 ± 0.092	2.031 ± 0.664	11.067 ± 1.223	20.060 ± 1.206
pHluorin-BACE1-mCherry	BafA1	0.191 ± 0.050	4.344 ± 1.022	5.200 ± 1.474	24.680 ± 3.153
BACE1-mCherry-pHluorin	BafA1	0.203 ± 0.069	4.099 ± 1.436	13.933 ± 2.631	35.640 ± 3.193
BACE1-mCherry-EGFP	BafA1	0.385 ± 0.082	1.918 ± 0.592	14.667 ± 1.799	36.153 ± 3.408

**Table 2:** Summary of BACE1 plasmids in response to CQ and BafA1 in cortical neurons.

Plasmid	Drug Treatment	Basal Ratio	Ratio Change (fold)	Response Time (min)	t_1/2_ (min)
pHluorin-BACE1-mCherry	CQ	0.192 ± 0.059	3.937 ± 1.455	2.400 ± 0.682	10.953 ± 3.596
BACE1-mCherry-pHluorin	CQ	0.172 ± 0.068	4.157 ± 1.443	5.333 ± 1.345	18.267 ± 3.625
pHluorin-BACE1-mCherry	BafA1	0.193 ± 0.066	4.570 ± 1.810	9.600 ± 3.334	30.333 ± 3.400
BACE1-mCherry-pHluorin	BafA1	0.189 ± 0.070	3.778 ± 1.137	15.267 ± 3.195	37.207 ± 4.881

## Supplementary Materials

The following are available online at https://www.mdpi.com/2073-4409/8/5/474/s1, Figure S1: Response of NLT cells expressing BACE1-mCherry-EGFP to CQ treatment; Figure S2: Response of NLT cells expressing pHluorin-BACE1-mCherry to LY2886721; Figure S3: Regulation of pHluorin-BACE1-mCherry and BACE1 activity by APP in NLT cells.

## Abbreviations

Aβ	β-amyloid
AD	Alzheimer’s disease
APP	amyloid precursor protein
APPswe	Swedish mutant APP
BACE1	β-site APP cleaving enzyme 1
BafA1	bafilomycin A1
BBS	α-bungarotoxin binding site
BFP	blue fluorescent protein
CMV	cytomegalovirus
CQ	chloroquine
DABCO	1,4-Diazabicyclo-octane
EGFP	enhanced green fluorescent protein
ER	Endoplasmic reticulum
FBS	fetal bovine serum
GFP	green fluorescent protein
HRP	horseradish peroxidase
IRES	internal ribosome entry site
IUE	in utero electroporation
NPCs	neural progenitor cells
NSCs	neural stem cells
PCR	polymerase chain reaction
PEI	polyethylenimine
PFA	paraformaldehyde
Pro	pro-peptide
proBACE1	immature precursor protein BACE1
PRR/ATP6AP2	(pro)renin receptor
PVA	poly(vinyl alcohol)
SP	signal peptide
TGN	trans-Golgi Networks
TMD	transmembrane domain
V-ATPase	vacuolar-type ATPase
