# Supplementary material for: pHluorin-BACE1-mCherry Acts as a Reporter for the Intracellular Distribution of Active BACE1 In Vitro and In Vivo
Source: Cells. 2019 May 17;8(5):474. doi: 10.3390/cells8050474 (PMC6562731; doi:10.3390/cells8050474)
Supplement: Supplementary file 1 [file cells-08-00474-s001.pdf]

Figure S1

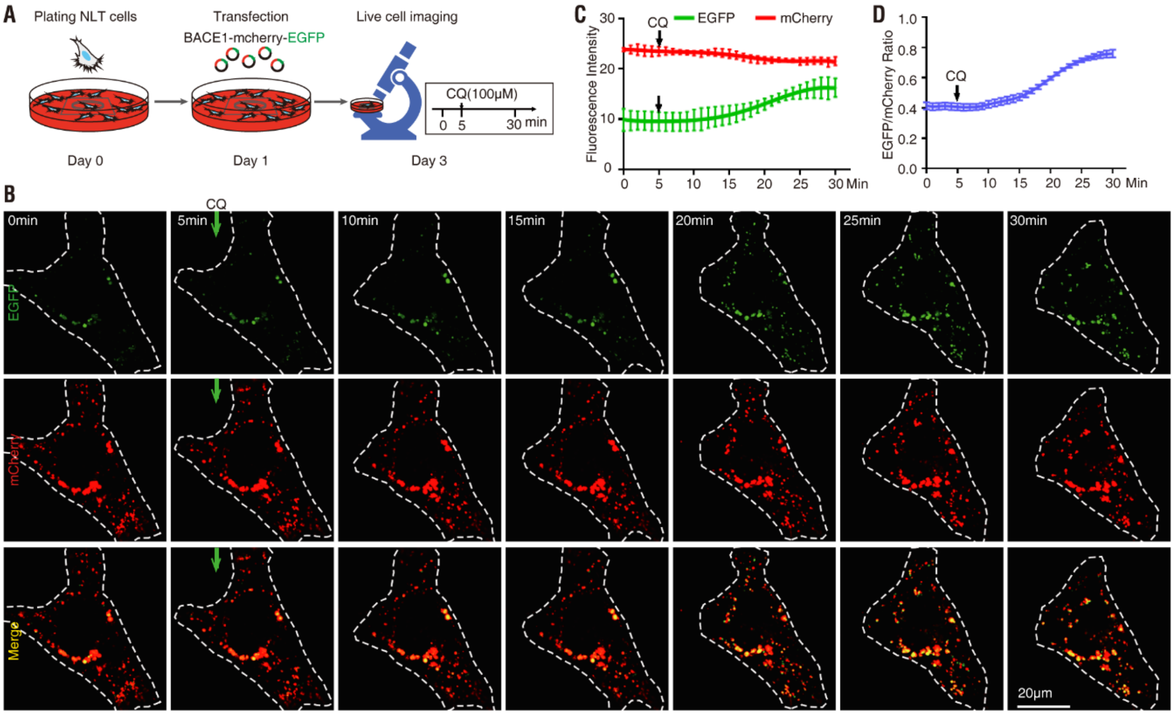

Figure S1. Response of NLT cells expressing BACE1-mCherry-EGFP to CQ treatment (**A**) Schematics of live cell imaging experimental procedures. NLT cells were plated on Day 0 and cotransfected with BACE1-mCherry-EGFP and BFP on Day 1. NLT cells expressing BACE1-mCherry-EGFP were imaged every one minute for 30 minutes on Day 3 and treated with 100 μM CQ after 5 minutes. (**B**) Representative images from live cell imaging in response to CQ. NLT cells expressing BACE1-mCherry-EGFP (red and green) were outlined according to BFP expression. Scale bar, 20 μm. (**C**) The fluorescence signal intensity of mCherry (red) and EGFP (green) in the outlined area was measured using ImageJ and plotted over time in response to CQ. Data were shown as mean ± SEM (n > 15 cells from three independent experiments). (**D**) Quantification analysis of the EGFP/mCherry ratio from C. Data was shown as mean ± SEM (n > 15 cells from three independent experiments).

Figure S2

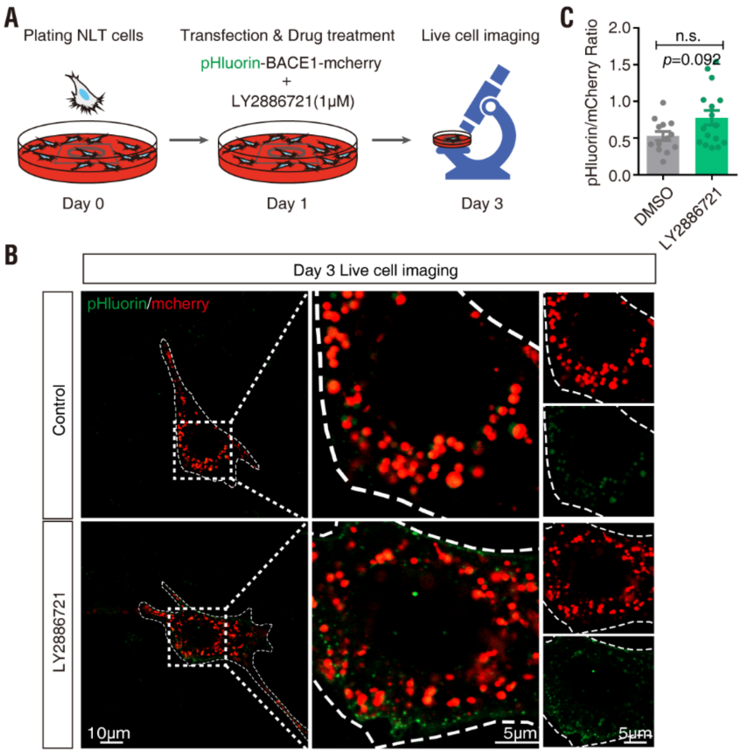

Figure S2. Response of NLT cells expressing pHluorin-BACE1-mCherry to LY2886721 (**A**) Schematics of live cell imaging experimental procedures. NLT cells were plated on Day 0 and cotransfected with pHluorin-BACE1-mCherry and BFP on Day 1. After 6 h, the culture medium was removed and replaced with 10% FBS DMEM containing 1 μM LY2886721. NLT cells expressing pHluorin-BACE1-mCherry were subjected to live cell imaging analysis on Day 3. (**B**) Representative images from live cell imaging in response to LY2886721. NLT cells expressing BACE1-mCherry-pHluorin (red and green) were outlined according to BFP expression. Scale bar, 10 μm/5 μm. (**C**) Quantification analysis of the pHluorin/mCherry ratio from B. Data was shown as mean ± SEM (n > 20 cells from three independent experiments). Significance was calculated with Student's t-test; n.s. $p > 0.05$.

Figure S3

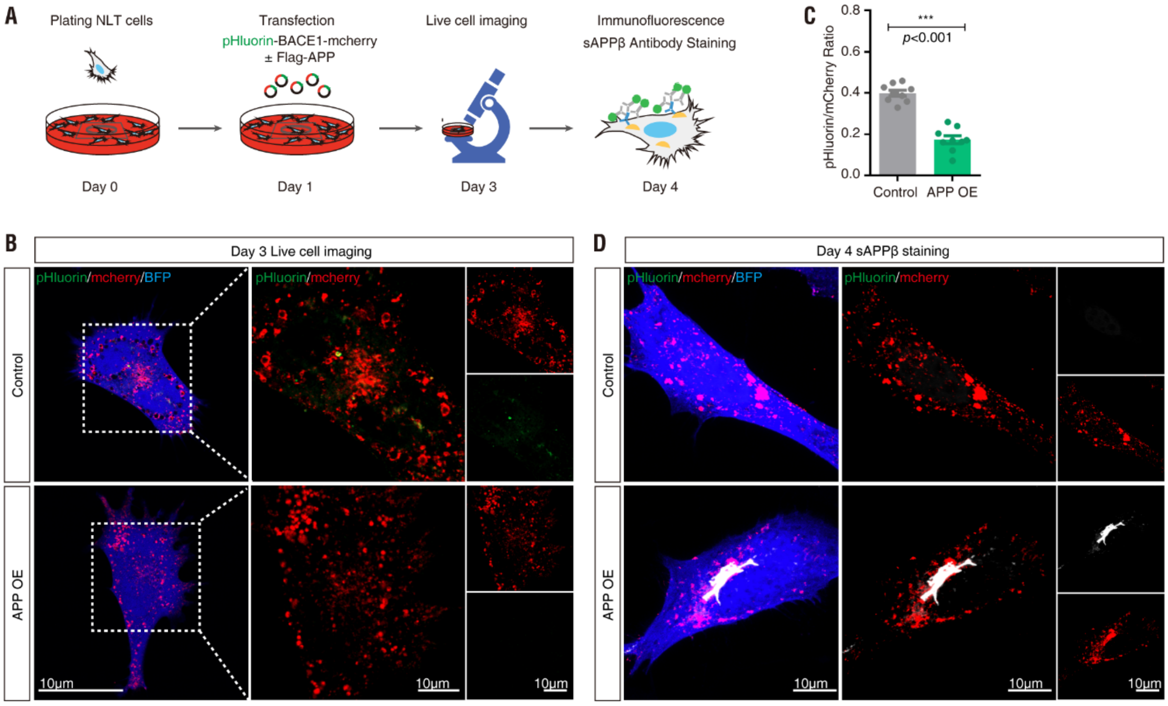

Figure S3. Regulation of pHluorin-BACE1-mCherry and BACE1 activity by APP in NLT cells (**A**) Schematics of live cell imaging experimental procedures. NLT cells were plated on Day 0 and cotransfected with pHluorin-BACE1-mCherry and BFP without Flag-APP (Control) or with Flag-APP (APP OE) on Day 1 and underwent live cell imaging on Day 3. Immunostaining of sAPPβ was performed on Day 4. (**B**) Confocal live cell imaging of transfected NLT cells on Day 3 was carried out, and representative images are shown. Scale bar, 10 μm. (**C**) Quantification analysis of the pHluorin/mCherry ratio from B. Data was shown as mean ± SEM (n > 10 cells from three independent experiments). (**D**), Immunostaining of sAPPβ in transfected NLT cells at DIV4 was carried out and representative images were shown. Scale bar, 10 μm. Significance was calculated with Student's t-test; *** $p < 0.001$.
